# Supplementary material for: Effectiveness and Safety of Ayurvedic Medicines in Type 2 Diabetes Mellitus Management: A Systematic Review and Meta-Analysis
Source: Front Pharmacol. 2022 Jun 8;13:821810. doi: 10.3389/fphar.2022.821810 (PMC9213670; doi:10.3389/fphar.2022.821810)
Supplement: Supplementary file 1 [file DataSheet1.zip › Supplementary Material/Forest and Funnel Plots/Punica granatum/HbA1c.pdf]

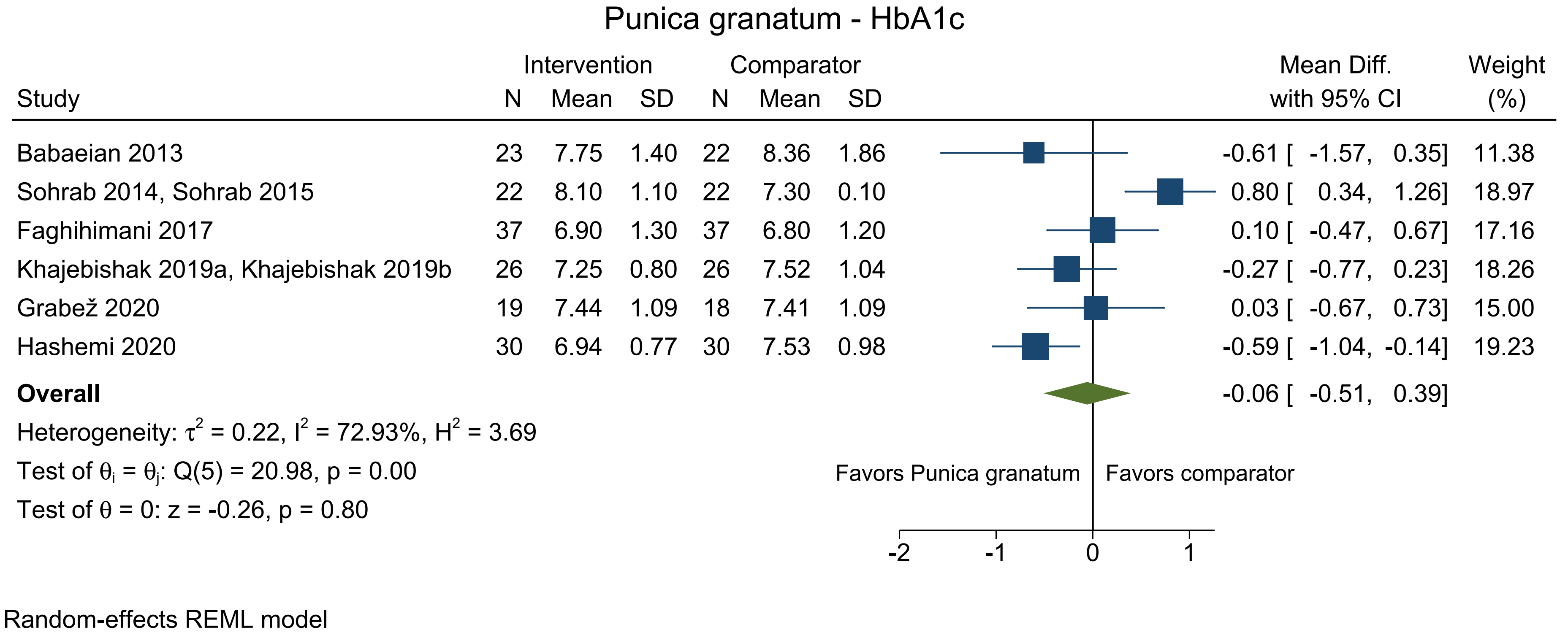
Punica granatum - HbA1c
Study
Intervention
N Mean SD
Comparator
N Mean SD
Mean Diff. with 95% CI
Weight (%)
Babaeian 2013 23 7.75 1.40 22 8.36 1.86 -0.61 [-1.57, 0.35] 11.38
Sohrab 2014, Sohrab 2015 22 8.10 1.10 22 7.30 0.10 0.80 [0.34, 1.26] 18.97
Faghihimani 2017 37 6.90 1.30 37 6.80 1.20 0.10 [-0.47, 0.67] 17.16
Khajebishak 2019a, Khajebishak 2019b 26 7.25 0.80 26 7.52 1.04 -0.27 [-0.77, 0.23] 18.26
Grabež 2020 19 7.44 1.09 18 7.41 1.09 0.03 [-0.67, 0.73] 15.00
Hashemi 2020 30 6.94 0.77 30 7.53 0.98 -0.59 [-1.04, -0.14] 19.23
Overall -0.06 [-0.51, 0.39]
Heterogeneity: τ² = 0.22, I² = 72.93%, H² = 3.69
Test of θi = θj: Q(5) = 20.98, p = 0.00
Test of θ = 0: z = -0.26, p = 0.80
Favors Punica granatum
Favors comparator
-2
-1
0
1
Random-effects REML model
